# Sporadic Shiga Toxin–Producing *Escherichia coli*–Associated Pediatric Hemolytic Uremic Syndrome, France, 2012–2021

**DOI:** 10.3201/eid2910.230382

**Published:** 2023-10

**Authors:** Gabrielle Jones, Patricia Mariani-Kurkdjian, Aurélie Cointe, Stéphane Bonacorsi, Sophie Lefèvre, François-Xavier Weill, Yann Le Strat

**Affiliations:** Santé publique France, Saint-Maurice, France (G. Jones, Y. Le Strat);; Centre Hospitalier Universitaire Robert Debré, Assistance publique–Hôpitaux de Paris, Paris, France (P. Mariani-Kurkdjian, A. Cointe, S. Bonacorsi);; Institut Pasteur, Université Paris-Cité, Paris (S. Lefèvre, F.-X. Weill)

**Keywords:** Escherichia coli, hemolytic uremic syndrome, Shiga toxin–producing Escherichia coli, STEC, epidemiologic surveillance, space-time clustering, food safety, bacteria, bacterial infections, France

## Abstract

Shiga toxin–producing *Escherichia coli*–associated pediatric hemolytic uremic syndrome (STEC-HUS) remains an important public health risk in France. Cases are primarily sporadic, and geographic heterogeneity has been observed in crude incidence rates. We conducted a retrospective study of 1,255 sporadic pediatric STEC-HUS cases reported during 2012–2021 to describe spatiotemporal dynamics and geographic patterns of higher STEC-HUS risk. Annual case notifications ranged from 109 to 163. Most cases (n = 780 [62%]) were in children <3 years of age. STEC serogroups O26, O80, and O157 accounted for 78% (559/717) of cases with serogroup data. We identified 13 significant space-time clusters and 3 major geographic zones of interest; areas of southeastern France were included in >5 annual space-time clusters. The results of this study have numerous implications for outbreak detection and investigation and research perspectives to improve knowledge of environmental risk factors associated with geographic disparities in STEC-HUS in France.

Shiga toxin–producing *Escherichia coli* (STEC) bacteria are responsible for a spectrum of disease, ranging from simple to bloody diarrhea, and pose increased risk for severe complications, including hemolytic uremic syndrome (HUS) in children <5 years of age and the elderly (*1*). Although STEC infections represent a global burden that is difficult to characterize, in part because of differences in diagnostic capacity and disease surveillance systems, an estimated 2.8 million STEC infections and 3,890 STEC-HUS cases occur annually worldwide (*2*). Estimated notification rates of STEC infection in Europe during 2017–2021 ranged from 1.6 to 2.4 cases/100,000 population (*3*). 

In France, STEC surveillance is conducted through voluntary clinical and microbiologic surveillance of HUS in children <15 years of age (*4*). Annual incidence rates for pediatric STEC-HUS in France remain relatively high, and in recent years have been close to estimated notification rates for all STEC infections in Europe (*5*). Since 1996, annual incidence rates have ranged from 0.6 to 1.5 cases/100,000 population (73 to 168 cases reported annually), and incidence has exceeded 4 cases/100,000 population in children <3 years of age (*5*). The primary serogroups identified in cases are O26, O80, and O157; an increase of serogroups O26 and O80 in the 2010s coincided with a decrease in O157 (*4*).

Ruminants are the primary reservoir, excreting STEC in their feces, thereby potentially contaminating food and their environment and posing a risk for STEC contamination in humans (*6*,*7*). Although STEC pose a substantial outbreak potential, most infections are sporadic; only 3% of cases reported in France during 2007–2016 were outbreak-associated (*4*). Determining the source of contamination for sporadic cases is difficult for numerous reasons, including limited epidemiologic data, the multiple potential sources of contamination, and gaps in knowledge about pathogen source–pathway interactions (*1*,*4*,*8*).

Annual incidence rates calculated from pediatric STEC-HUS surveillance in France show regional variations. Space–time cluster detection methods, which can be applied to epidemiologic surveillance for outbreak detection, are also of interest for studying sporadic infectious disease cases. Indeed, several recent studies illustrate such approaches for describing spatiotemporal disease patterns and identifying recurrent geographic clusters representing differences in baseline disease risk, including STEC infection, cryptosporidiosis, and salmonellosis (*8*–*12*).

The first objective of our study was to describe temporal trends and geographic distribution of sporadic pediatric STEC-HUS cases in France over a 10-year period. The second objective was to identify space–time clusters and describe geographic patterns of significantly higher risk for sporadic STEC-HUS at a fine geographic scale. Such data are crucial for enabling epidemiologic surveillance, including assessment of clusters requiring epidemiologic investigations. In addition, robust, statistically significant data identifying geographic disparities at a fine scale open perspectives for further research aiming to better understand potential environmental, sociodemographic, and even behavioral factors associated with observed differences.

## Methods

### Data Sources and Processing

Cases of suspected STEC-HUS in children <15 years of age are reported to Santé publique France (France’s national public health agency) according to previously described clinical criteria (*4*). Surveillance data include demographic information (age, sex, and postal codes of residence and temporary stay), clinical data (diarrhea, date of diarrhea onset, and date of HUS diagnosis), results of stool analysis, and limited epidemiologic information (outbreak-related [community, childcare, or family setting] and primary at-risk exposures). In accordance with data protection procedures defined by France’s oversight authority for privacy and data protection (Commission Nationale de l’Informatique et des Libertés), age, postal codes, dates of diarrhea onset, and HUS diagnosis are conserved indefinitely for surveillance purposes.

Microbiologic STEC surveillance is voluntary and coordinated by the National Reference Center (NRC) for *Escherichia coli*, *Salmonella* and *Shigella* (Institut Pasteur, Paris, France) and its associated laboratory (University Hospital Robert Debré, Assistance publique–Hôpitaux de Paris, Paris) (*4*). Over the entire study period, NRC conducted PCR testing of stool samples to detect virulence genes (*stx1, stx2, eae,* and *ehxA*) and O-antigen biosynthesis genes of the 10 most frequent STEC serogroups identified in France (O157, O26, O103, O145, O91, O121, O104, O55, O111, and O80 [in 2013]). NRC performed culture on all *stx*- or *eae*-positive stools. Serogrouping methods evolved over the study period (*13*,*14*) (Appendix).

We identified 1,419 notified pediatric STEC-HUS cases with symptom onset during January 1, 2012–December 31, 2021, from surveillance data. For cases with no diarrhea, we used date of HUS diagnosis. We defined sporadic cases as those having no documented epidemiologic link to other confirmed STEC or STEC-HUS cases. For cases of person-to-person transmission in a family or childcare setting, the first case was retained for analysis because the index case is considered a sporadic case potentially associated with geographically specific exposures. We excluded from analysis all foodborne or environmentally associated outbreak cases and cases in patients with reported international travel during the entire exposure period. We were able to link cases with whole-genome sequencing (WGS) data for 2018–2021. We retained in the analysis cases belonging to a WGS-linked cluster but with no common source of infection suspected from epidemiologic investigations. Also, we excluded cases with no postal code available because they could not be geocoded. Postal codes of temporary stay correspond to locations visited in France in the week before diarrhea onset. Furthermore, we restricted analyses to cases reported from mainland France. In total, we excluded 164 (12%) of 1,419 reported cases. 

We used population data for children <15 years of age available for the period 2012–2018 from the National Institute for Statistics and Economic Studies. For the remaining study period of 2019–2021, we used the most recent census data available (2018). Geocoding of cases used postal code of residence or of temporary stay when available (n = 5).

We obtained all study data from anonymous surveillance data conserved by Santé publique France according to an ongoing authorization by the Commission Nationale de l’Informatique et des Libertés. No additional ethics approval was required.

### Temporal and Spatial Analyses

We described temporal and spatial distributions for all cases and by subgroups for 2 variables of epidemiologic importance, age of patient and STEC serogroup. We defined 6 age groups on the basis of observed distribution in STEC-HUS incidence from surveillance data: <1 year, 1 year, 2 years, 3–4 years, 5–9 years, 10–14 years. Those groups were also statistically pertinent for calculation of standardized incidence rates. We conducted serogroup-specific subgroup analyses for the 3 most frequently identified serogroups in pediatric STEC-HUS cases in France, O26, O80, and O157 (top-3 serogroups). For other serogroups, the number of isolates was insufficient for subgroup analysis (mean <5 isolates/year). We classified cases into 4 groups (Appendix): top-3 serogroups, other serogroup, no serogrouping, and ungroupable. We retained for overall analysis children with HUS but no microbiologic confirmation of STEC infection on the basis of clinical criteria (diarrhea before HUS) and the fact that in young children, HUS is largely associated with STEC infection (*4*).

We described temporal trends and spatial distribution by using age-standardized incidence rates calculated by the direct method. We represented spatial distribution at the administrative department level (n = 95). By using a moving average method, we decomposed monthly incidence rates into trend, season, and residual components through the decompose function with an additive model using R version 4.2 (The R Foundation for Statistical Computing, https://www.r-project.org).

### Space-time Scan Statistics

We conducted a retrospective analysis for space-time cluster detection using SaTScan version 9.7 (https://www.satscan.org), including all cases and for each of the top-3 serogroups. We geocoded all cases to the établissement public de cooperation intercommunale (EPCI), a geographic unit that groups municipalities on the basis of administrative criteria (1,233 EPCI are in mainland France). The scan statistic tests whether cases are randomly distributed over space and time and identifies clusters for which a significant difference exists in the number of observed cases compared with expected cases (*15*,*16*). For each cluster, the main outputs were geographic information (coordinates of the center of the circle and radius); cluster time interval by date, population size, and number of observed and expected cases; relative risk; and p value. We considered clusters significant at p<0.05.

We used a discrete Poisson model because of high spatial resolution of the geographic unit of analysis and the rare occurrence of STEC-HUS cases (<1 case for most ECPIs). We included age as a covariable for all analyses. We chose different tuning parameters to assess their effect on results. On the basis of the sensitivity analysis, we restricted the maximum radius of the circular scan window to 100 km and maximum geographic cluster size to 10% of the population at risk. We set the time precision unit to days with a maximum cluster duration of 90 days to account for observed seasonality of STEC-HUS (*8*,*11*). We retained a minimum number of cases (cluster restriction) of 2. We performed space–time scans over the 10-year study period and annually. On the basis of the results of annual space–time scans, we calculated the cluster recurrence index developed by Boudou et al. to identify geographic areas where significant clusters occurred at least once during the study period (*10*). We conducted all statistical analyses by using R version 4.2 and SaTScan version 9.7 (*17*).

## Results

### Number of Cases over Time

**Figure 1:**
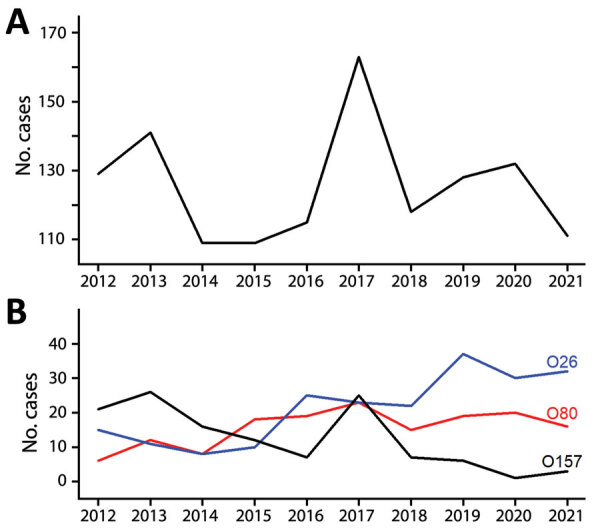
Annual reported number of sporadic Shiga toxin–producing *Escherichia coli*–associated pediatric hemolytic uremic syndrome cases, France, 2012–2021. A) All cases; B) cases of infection with serogroups O26, O80, and O157.

A total of 1,255 sporadic pediatric STEC-HUS cases were reported during January 2012–December 2021; the annual number of cases ranged from 109 in 2014 and in 2015 to 163 in 2017 (Figure 1). Cases were distributed in 542 (44%) of 1,233 EPCIs. A sample was sent to NRC for 1,132 (90%) cases, and 717 (63%) of those had a STEC serogroup identified (Appendix Table 1, Figure 1). The top-3 serogroups accounted for 78% (559/717) of cases with serogroup data: O26, 228 cases (41%); O80, 149 cases (27%); and O157, 182 cases (33%). Approximately 23% (255/1,132) of cases were ungroupable. The 283 (23%) cases with no serogrouping consisted of 123 with no sample sent to NRC (24 [20%] with reported PCR detection of *stx* at the notifying hospital) and 160 that were negative at NRC (PCR *stx* or serologic tests). For the 259 cases with no *stx* detection or negative serologic tests, 95% (247) were in patients who had diarrhea before HUS.

**Figure 2:**
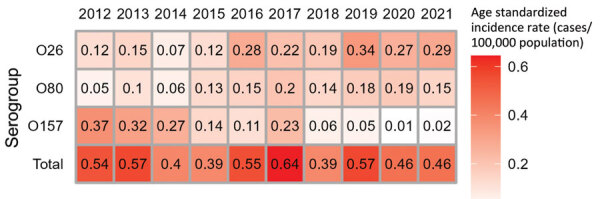
Age-standardized annual incidence rates of reported sporadic Shiga toxin–producing *Escherichia coli*–associated pediatric hemolytic uremic syndrome cases, for all cases and cases of infection with serogroups O26, O80, and O157, France, 2012–2021.

We observed no overall trend in the annual number of sporadic cases notified or in age-standardized annual incidence rates during 2012–2021. However, for STEC O26 and O80, we observed a significant increasing trend, in parallel to a significant decreasing trend for O157 (Figures 1, 2).

**Figure 3:**
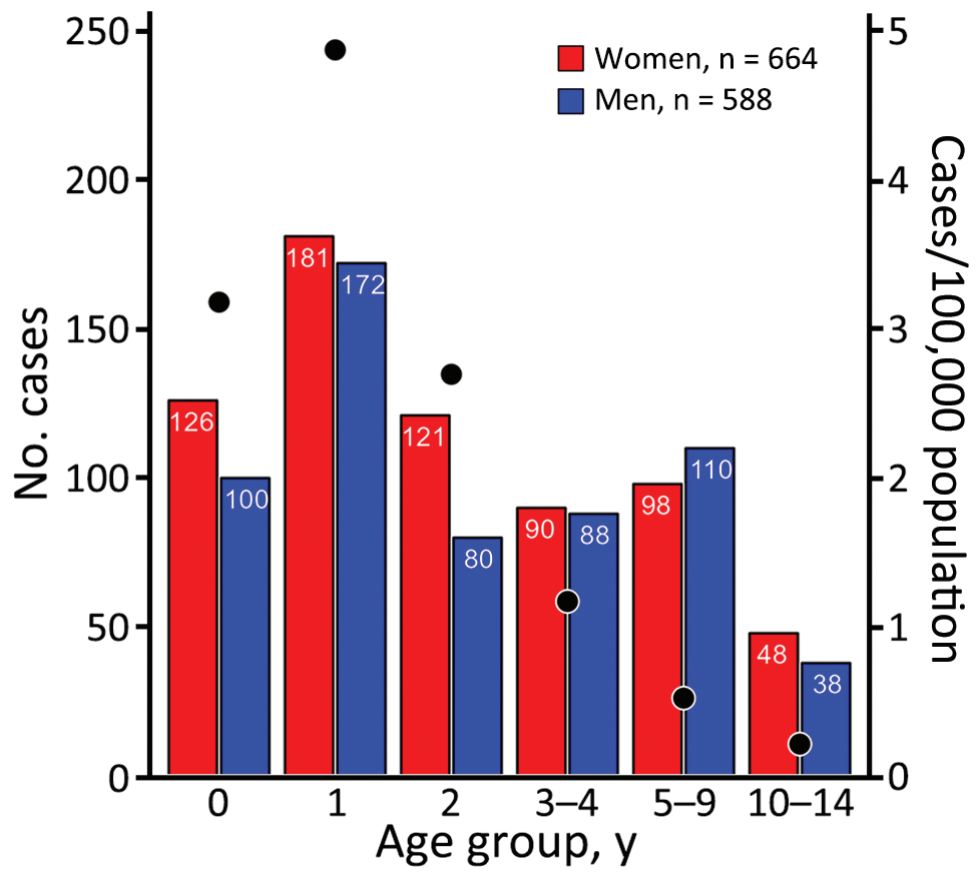
Number of reported sporadic Shiga toxin–producing *Escherichia coli*–associated pediatric hemolytic uremic syndrome cases, by age group and sex, and incidence rate, by age group (black dots), France, 2012–2021. Data were missing on sex for 3 cases (1 case each in patients <1 year, 3 years, and 4 years of age).

The proportions of female and male case-patients were comparable over the entire study period (Figure 3). Most cases (780/1,255 [62%]) were in patients <3 years of age. Incidence rate varied by age group; the highest incidence was in children 1–2 years of age (4.9 cases/100,000 population) (Figure 3). Incidence rate by age varied slightly by serogroup; the highest incidence was in younger age groups (1–2 years) for STEC O80 (0.9 cases/100,000 population) and STEC O26 (1 case/100,000 population), compared with incidence in that age group for STEC O157 (0.2 cases/100,000 population).

### Temporal and Spatial Distribution of Incidence Rates

Seasonal decomposition of all sporadic STEC-HUS cases confirmed seasonality with a distinct annual peak (Appendix Figure 2). Annual peaks also were observed for the top-3 serogroups but with associated secondary and tertiary peaks between the primary seasonal peaks.

**Figure 4:**
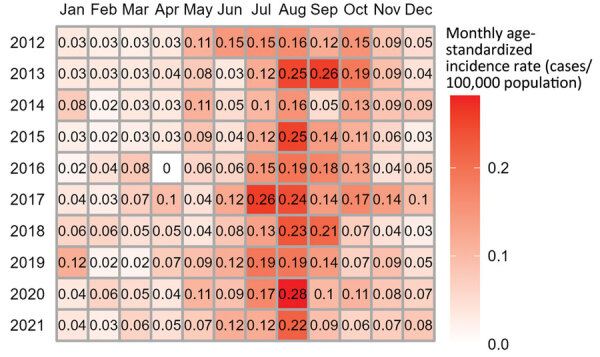
Monthly age-standardized incidence rates of reported sporadic Shiga toxin–producing *Escherichia coli*–associated pediatric hemolytic uremic syndrome cases, France, 2012–2021.

**Figure 5:**
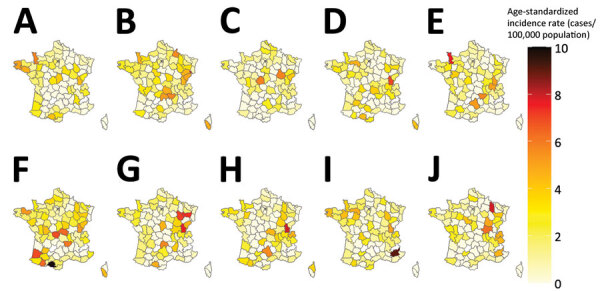
Geographic distribution of age-standardized incidence rates of all reported sporadic Shiga toxin–producing *Escherichia coli*–associated pediatric hemolytic uremic syndrome cases, France, 2012–2021. A) 2012; B) 2013; C) 2014; D) 2015; E) 2016; F) 2017; G) 2018; H) 2019; I) 2020; J) 2021.

Seasonality was observed in monthly age-standardized incidence rates for each year of the study period. The highest incidence occurred in summer and early fall (July–October) (Figure 4). No clear spatial trends in annual age-standardized incidence rates were evident during the study period. The departments with the highest incidence rates varied from 1 year to the next (Figure 5).

**Figure 6:**
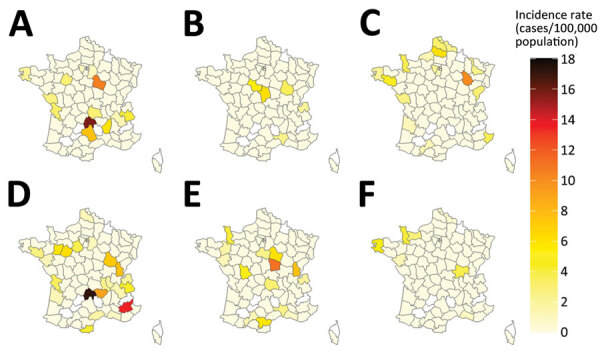
Geographic distribution of age-standardized incidence rates of reported sporadic Shiga toxin–producing *Escherichia coli*–associated pediatric hemolytic uremic syndrome cases caused by serogroups O26, O80, and O157, France, 2012–2021. A–C) Serogroups O26 (A), O80 (B), and O157 (C) during 2012–2016. D–F) Serogroups O26 (D), O80 (E), and O157 (F) during 2017–2021.

We described spatial distribution for the top-3 serogroups over 2 periods (2012–2016 and 2017–2021) to account for a smaller number of cases. For STEC O26, we observed a geographic extension of cases when comparing the 2012–2016 period to the 2017–2021 period (Figure 6). We observed a similar evolution for STEC O80. For STEC O26 and STEC O80, departments in the eastern half of France had slightly higher incidence rates. For STEC O157, the highest incidence rates were primarily in departments in northwest France, regardless of the period.

### Space–Time Scanning

**Figure 7:**
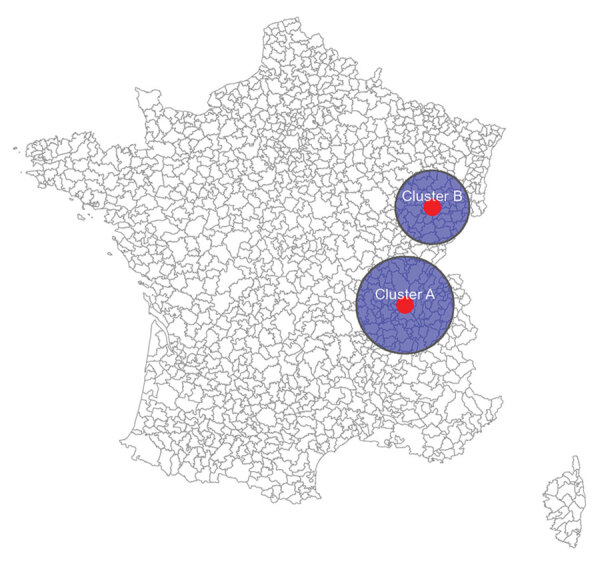
Significant clusters detected by space–time scanning of all reported sporadic Shiga toxin–producing *Escherichia coli*–associated pediatric hemolytic uremic syndrome cases, France, 2012–2021.

Space–time scanning of all cases over the entire 10-year period identified 2 significant clusters occurring in 2019 and 2013, primarily in 2 regions in eastern France (Table 1; Figure 7). Scanning over the 10-year period by serogroup identified 2 significant clusters: STEC O26 (2019, southeast France) and STEC O80 (2017, northeast France) (Appendix Table 2, Figure 3). WGS data available for the isolates within the 2019 O26 space-time cluster identified 3 WGS-linked clusters of 2 isolates each (EnteroBase [https://enterobase.readthedocs.io] cgMLST scheme, HC5 level) (*14*). Short-read sequences are available in EnteroBase (identification nos. 201904732, 201904733, 201905626, 201905634, 201907203, and 201908310) and in the European Nucleotide Archive (study no. PRJEB50273). For each pair of WGS-linked isolates, epidemiologic investigations did not identify a suspected common source of infection.

**Table 1:** Characteristics of significant clusters detected by space–time scanning of all reported sporadic Shiga toxin–producing *Escherichia coli*–associated pediatric hemolytic uremic syndrome cases, France, 2012–2021

Cluster ID	Start date	End date	Radius, km	Population	Observed no.	Expected no.	Relative risk	p value
2019 cluster A	2019 Jun	2019 Jun	96	1,073,339	20	2.8	7.2	0.00159
2013 cluster B	2013 Aug	2013 Oct	73	217,195	9	0.43	21.1	0.024

*ID, identification.

**Figure 8:**
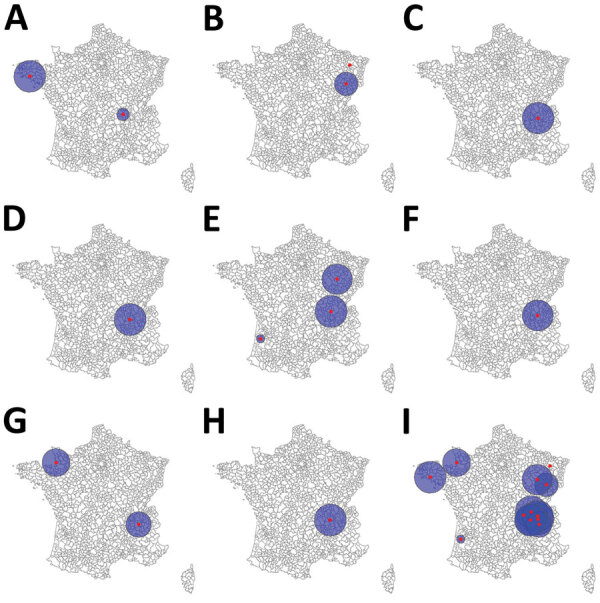
Significant clusters detected by annual space–time scanning of all reported sporadic Shiga toxin–producing *Escherichia coli*–associated pediatric hemolytic uremic syndrome cases, France, 2012–2021. A) 2012; B) 2013; C) 2015; D) 2016; E) 2018; F) 2019; G) 2020; H) 2021; I) 2012–2021.

Annual space–time scanning identified 13 significant space–time clusters (Table 2; Figure 8). We identified >1 significant cluster for each year, with the exception of 2014 and 2017, and detected a maximum of 3 significant clusters in 2018. Median cluster size was 10 cases (range 2–20 cases). Clusters occurred exclusively in the period of June–November, and most clusters corresponded to the seasonal peak observed in STEC-HUS notifications during July–October. Annual scanning by serogroup did not identify any significant space–time clusters.

**Table 2:** Characteristics of significant clusters detected each year by space–time scanning of all reported sporadic Shiga toxin–producing *Escherichia coli*–associated pediatric hemolytic uremic syndrome cases, France, 2012–2021

Cluster ID	Start date	End date	Radius, km	Population	Observed no.	Expected no.	Relative risk	p value
2012 1	2012 Aug	2012 Oct	98	343,365	10	0.79	13.7	0.024
2012 2	2012 Jul	2012 Aug	38	111,646	6	0.15	41.7	0.024
2013 1	2013 Aug	2013 Oct	73	223,770	9	0.47	20.18	0.00748
2013 2	2013 Nov	2013 Nov	0	1,278	2	0.0002	10013.3	0.031
2015 1	2015 Aug	2015 Oct	98	1,090,917	18	2	10.56	0.0000253
2016 1	2016 Jun	2016 Sep	99	1,001,768	17	2.39	8.16	0.00215
2018 1	2018 Jun	2018 Sep	94	253,429	10	0.58	18.61	0.00196
2018 2	2018 Sep	2018 Sep	25	37,752	3	0.0058	527.19	0.031
2018 3	2018 Jul	2018 Sep	99	926,727	11	1.15	10.45	0.044
2019 1	2019 Jun	2019 Sep	96	1,082,597	20	2.94	7.88	0.000202
2020 1	2020 Jun	2020 Aug	86	333,046	10	0.75	14.3	0.017
2020 2	2020 Aug	2020 Aug	79	858,465	8	0.38	22.26	0.017
2021 1	2021 Jul	2021 Aug	99	999,909	10	0.66	16.46	0.00501

*ID, identification.

**Figure 9:**
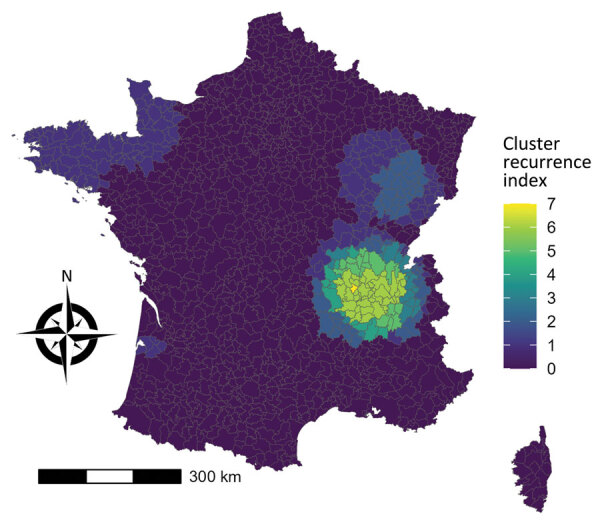
Cluster recurrence index of all reported sporadic Shiga toxin–producing *Escherichia coli*–associated pediatric hemolytic uremic syndrome cases, France, 2012–2021.

The cluster recurrence index ranged from zero to 7 and identified an area of particularly high recurrence in southeast France; certain EPCIs were included in >5 clusters during the study period (Figure 9). Two additional major geographic zones were identified as hotspots, although to a lesser degree, in the northwest and northeast.

## Discussion

During 2012–2021, a mean of 125 cases of sporadic pediatric STEC-HUS were reported in France annually. Incidence was highest in children <3 years of age, and particularly in children 1–2 years of age (an incidence 1.7 times greater than that observed in children <1 year of age and 2 times greater than that observed in children 2–3 years of age). Analysis of temporal trends by serogroup showed the increase of HUS-associated STEC O26 and STEC O80 in parallel to the decrease in STEC O157. Similar trends have been observed in other countries of Europe (*3*,*18*). Department-level age-standardized incidence rates showed geographic heterogeneity and no clear patterns when considering all STEC-HUS cases. Subgroup analysis showed the geographic extension of STEC O26 and STEC 80.

Annual space–time scanning identified 13 significant clusters occurring over a 10-year period and confirmed geographic disparities in cases. Significant clusters occurred almost exclusively during the seasonal peak of STEC-HUS observed during July–October. Application of the cluster recurrence index identified several geographic zones of interest, in particular southeast France, with certain EPCIs included in >5 recurrent space–time clusters. This zone is in the second most densely populated region in France and includes a major city, Lyon, but also rural areas and high cattle density (*19*). To a lesser extent, we identified notable space–time clusters in regions in northwest and northeast France (1–2 clusters during the study period). In contrast to the southeast, those regions are less populated and have smaller population centers. As in southeast France, cattle density is higher than elsewhere in France (*19*).

One limitation of our analysis is that we hypothesized that sensitivity of reporting was constant over space and time for the 10-year study period. Previous studies estimated sensitivity of reporting at 66% during 2002–2003 and 85% during 2016–2017 (*20*; Santé publique France, unpub. data). Variations may have occurred in the sensitivity of reporting over time or between regions. In particular, the occurrence of outbreaks might improve reporting because of greater awareness among clinicians after outbreak events. Of note, several foodborne STEC-HUS outbreaks with wide media coverage occurred during 2018–2019 (*21*–*23*). However, several aspects of the surveillance system limit potential bias. In particular, the surveillance system has been in place since 1996 and has a stable network of specialized hospital units, enabling Santé publique France to be in regular contact with clinicians and maintain a high level of awareness for reporting of cases. In addition, an annual reminder is sent to all participating hospital units asking them to notify any cases missed during the previous year. Therefore, our retrospective analysis is less likely to be affected by heterogeneous reporting.

Moreover, space–time analysis relied on EPCIs under the hypothesis that the at-risk STEC exposure occurred in the place of residence or in another reported place of exposure in France. However, case-patients may have more limited geographic movements corresponding to at-risk exposures that are not documented in surveillance data. In this case, the documented EPCI would not correspond to the actual geographic area of at-risk exposure, but because EPCIs corresponds to grouped municipalities, this factor should limit the potential bias.

Furthermore, microbiologic data was limited for some cases, and microbiologic surveillance evolved over the 10-year study period. Including cases based solely on clinical criteria may have resulted in non–STEC-related HUS. However, almost all case-patients had diarrhea before HUS, and detection of *stx* genes may be hindered by several factors, including antibiotic treatment and the delay between diarrhea onset and sampling. Because postdiarrheal HUS in young children is largely attributable to STEC infection, we considered the limited risk for including non–STEC-associated cases (*4*). In addition, the power of the serogroup analysis is probably reduced by the fact that not all reported cases had a stool sample sent to NRC for analysis or that no serogroup was identified for some cases. Introducing routine WGS data into epidemiologic surveillance in 2017 has improved characterization of isolates and detection of related strains and of potential outbreaks (e.g., those with diffuse or fewer cases) (*24*–*26*). As a result, the capacity to detect outbreak cases from pediatric STEC-HUS surveillance in France evolved, and during 2012–2016, cases belonging to smaller outbreaks possibly were not detected and would be misclassified as sporadic cases in our study. A possible result of such misclassification could be a greater number of space-time clusters identified during this period compared with 2017–2021. However, this difference did not occur; the number of space-time clusters per year is comparable between the periods before and after introduction of WGS. Also, although WGS results can suggest potential links between isolates, Besser et al. discussed the importance of epidemiologic investigations in the assessment of WGS-linked isolates (*24*), and the existence of a common source cannot be assumed. We therefore excluded WGS-linked isolates only if epidemiologic investigation suspected a common source of infection.

Space-time cluster detection is sensitive to the selection of parameters in SaTScan. Chen et al. discussed the challenges and potential effect of those parameter choices (*27*), and published studies offer limited guidance for parameter choice, in particular given differences in characteristics of diseases and study populations. To limit the influence of parameter choice on results, we conducted a sensitivity analysis, and our selected parameters produced robust results even when individual parameters, such as maximum population at risk, varied. We considered a maximum radius of 100 km for the scanning window as relevant for this study because the underlying hypothesis is that higher relative risk for sporadic STEC-HUS in certain geographic areas may be caused by unidentified environmental factors. Therefore, identifying clusters with unlimited radii is not epidemiologically relevant.

Our study adds to an existing body of research demonstrating the effect of applying scan statistics to describe spatiotemporal dynamics of sporadic disease, even for more rare occurrences. Our results provide important insight into the epidemiologic context and have implications for outbreak detection and investigation and for research perspectives to improve knowledge of risk factors associated with geographic disparities in disease. The identification of several geographic areas with recurring clusters of sporadic STEC-HUS confirms statistically, and at a much finer geographic scale, previous observations of disparities in regional incidence of pediatric STEC-HUS in France (*4*,*19*). Taking into account geographic differences is relevant to analysis of surveillance data for outbreak detection purposes, in particular for evaluating epidemiologic signals and the decision to initiate investigations. The different geographic relative risks identified in this study will be integrated into SaTScan as part of ongoing research into its application for outbreak detection in France (*28*). Compared with WGS, statistical space-time cluster detection provides a reactive approach that can be applied to case notification data before WGS data are available (e.g., delays of ≈3 weeks in France) or in absence of strain isolation.

Our study also provides the necessary data and justification for further research on geographic factors associated with a higher baseline risk for STEC-HUS in France. Ecologic studies conducted in several countries using STEC surveillance data have identified significant associations with ruminant density, rural classification, and water sources, in particular private well usage (*9*,*29*–*32*). The findings of a study in France by Haus-Cheymol et al. suggested an association between pediatric STEC-HUS incidence and dairy cattle and calf density. The described geographic distribution of higher dairy cattle density in that study overlaps in part with the higher-risk geographic zones identified in our study in northwest and eastern France (*19*). However, the study merits an update because it is from the early 2000s, is limited to a more macroscopic geographic level, and covers a period before several observed evolutions in STEC epidemiology in France.

Our analysis also identified significant, recurring, space-time clusters consisting of cases with different serogroups. This finding suggests conditions favorable for STEC transmission that may contribute to higher risk for STEC-HUS, including geographic differences that could influence STEC risk because of different patterns of food and environmental exposures through a range of transmission pathways. We plan to use our results in further studies aimed at exploring the association with environmental parameters potentially underlying STEC-HUS risk in France. Conducting such a study at a finer geographic scale would aim to provide improved insight for public health professionals to target and adapt public health interventions, including communication with the general population, aimed at STEC prevention.

Appendix Additional information about sporadic Shiga toxin–producing *Escherichia coli*–associated pediatric hemolytic uremic syndrome, France, 2012–2021.
