# Supplementary material for: Sporadic Shiga Toxin–Producing Escherichia coli–Associated Pediatric Hemolytic Uremic Syndrome, France, 2012–2021
Source: Emerg Infect Dis. 2023 Oct;29(10):2054–64. doi: 10.3201/eid2910.230382 (PMC10521606; doi:10.3201/eid2910.230382)
Supplement: Appendix — Additional information about sporadic Shiga toxin–producing Escherichia coli–associated pediatric hemolytic uremic syndrome, France, 2012–2021. [file 23-0382-Techapp-s1.pdf]

Article DOI: https://doi.org/10.3201/eid2910.230382

Sporadic Shiga Toxin–Producing *Escherichia coli*–Associated Pediatric Hemolytic Uremic Syndrome, France, 2012–2021

Appendix

Methods

Microbiologic STEC Surveillance

After initial PCR and culture, isolated strains underwent characterization of serogroup and virulence profile by PCR (limited to the 10 major serogroups) as well as antimicrobial resistance testing. From 2012 to early 2017, serogroup determination was also performed by O-serotyping and if necessary by restriction of the amplified O-antigen gene cluster (*rfb*-RFLP). For a subset of cases with no stool sample or absence of STEC isolation, serologic testing was also performed for eight major serogroups (O26, O55, O91, O103, O111, O128, O145, O157 and O104) (*1*). Since early 2017, the NRC-ESS routinely performs real-time whole genome sequencing (WGS) on all STEC isolates, including *in silico* serotyping, determination of virulence genes and determination of cluster affiliation using core genome multilocus sequencing typing (cgMLST) (*2*).

Classification of Subgroups

We classified cases into four groups according to the following diagnostic method criteria: 1) Top-3 serogroups (PCR/O-serotyping of isolated strains or serology), 2) other serogroup (PCR/O-serotyping of isolated strains or serology), 3) no serogrouping (STEC not confirmed at NRC: no sample at NRC, or negative at NRC for PCR of *stx* gene(s) or serology), and 4) ungroupable (no strain isolation, but PCR amplification of *stx* gene(s) and absence of amplification of the O-antigen of the 10 principal serogroups, or amplification of O-antigen for two serogroups simultaneously).

References

1. King LA, Nogareda F, Weill F-X, Mariani-Kurkdjian P, Loukiadis E, Gault G, et al. Outbreak of Shiga toxin–producing *Escherichia coli* O104:H4 associated with organic fenugreek sprouts, France, June 2011. Clin Infect Dis. 2012;54:1588–94. PubMed https://doi.org/10.1093/cid/cis255

2. Zhou Z, Alikhan NF, Mohamed K, Fan Y, Achtman M; Agama Study Group. The EnteroBase user's guide, with case studies on *Salmonella* transmissions, *Yersinia pestis* phylogeny, and *Escherichia* core genomic diversity. Genome Res. 2020;30:138–52. PubMed https://doi.org/10.1101/gr.251678.119

Appendix Table 1. Case classification according to diagnostic data available of all sporadic Shiga toxin-producing *Escherichia coli*-associated pediatric hemolytic uremic syndrome cases reported in France from 2012–2021

Characteristic	Total	O26	O80	O157	Other	Ungroupable	Not serotyped
STEC isolation at NRC							
Serogrouping by PCR	560	163	131	85	102	79	–
Serogrouping by other methods*	69	24	1	28	16	–	–
PCR testing for STEC in stool at NRC/no STEC isolated							
stx negative	79	–	–	–	–	–	79
stx positive, no serogroup identified	146	–	–	–	–	146	–
stx positive, serogroup identified	102	20	17	10	31	24†	–
Serology at NRC (no stool sample available)							
Serology negative	81	–	–	–	–	–	81
Serology positive	95	21	–	59	9	6	–
No sample sent to NRC	123	–	–	–	–	–	123 ‡
Total	1255	228	149	182	158	255	283

NRC: National reference center; -: no isolates.
*O-serotyping and PCR-RFLP during the first part of the study (2012–2017).
† At least two serogroups identified.
‡24 isolates *stx*+ at notifying hospital laboratory.

Appendix Table 2. Characteristics of significant clusters detected by space-time scanning by serogroup* of sporadic Shiga toxin-producing *Escherichia coli*-associated pediatric hemolytic uremic syndrome cases reported in France from 2012–2021

Cluster ID	Start date (mm/yyyy)	End date (mm/yyyy)	Radius (km)	Population	Observed number	Expected number	Relative risk	p-value
2019 O26	06/2019	06/2019	85	880,223	9	0.41	22.7	0.01
2017 O80	07/2017	08/2017	54	110,417	3	0.0033	922.5	0.028

*Serogroups O26, O80, O157.

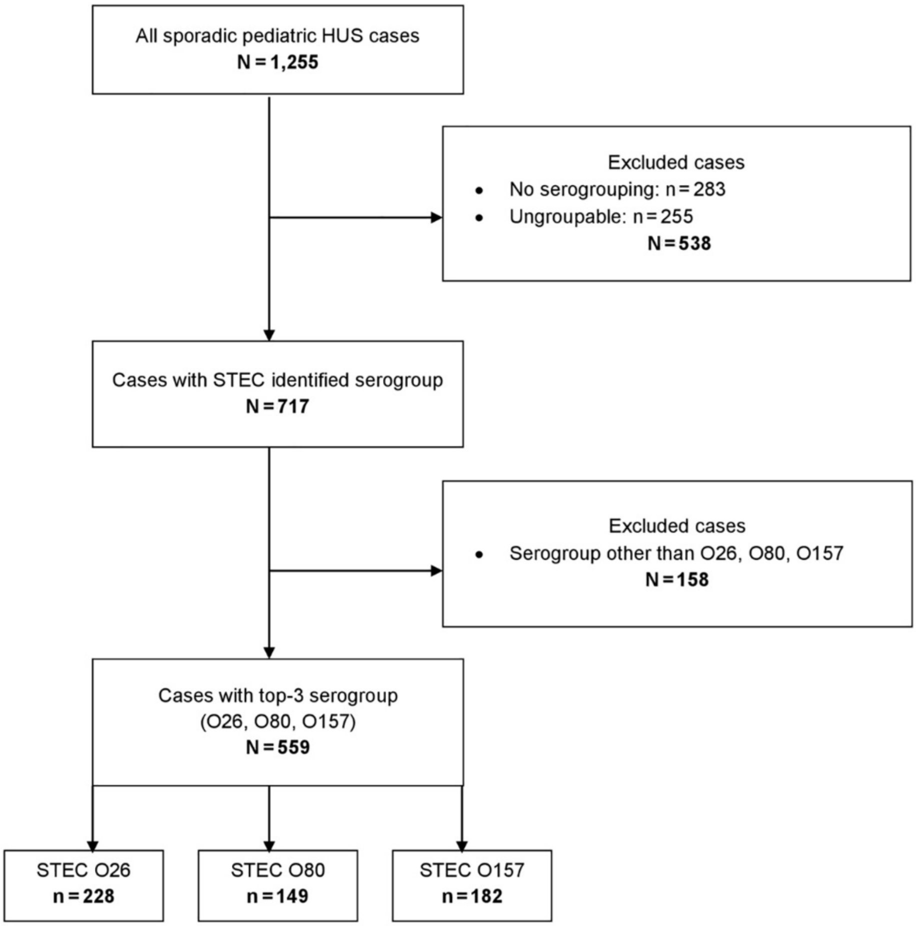

Appendix Figure 1. Flowchart of case classification by serogroup of all sporadic Shiga toxin-producing *Escherichia coli*–associated pediatric hemolytic uremic syndrome cases reported in France from 2012–2021.

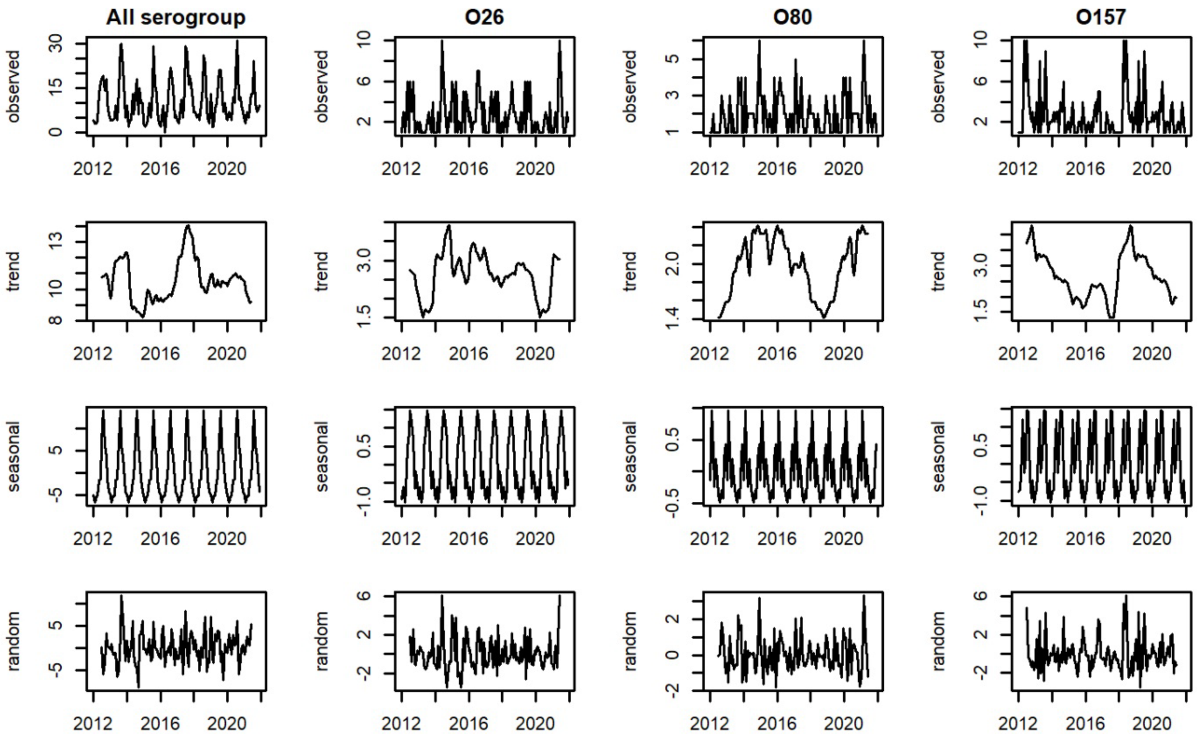

Appendix Figure 2. Seasonal decomposition of 10-year trends for sporadic Shiga toxin-producing *Escherichia coli*–associated pediatric hemolytic uremic syndrome cases reported in France from 2012–2021, for all serogroups and by serogroup (O26, O80, O157).

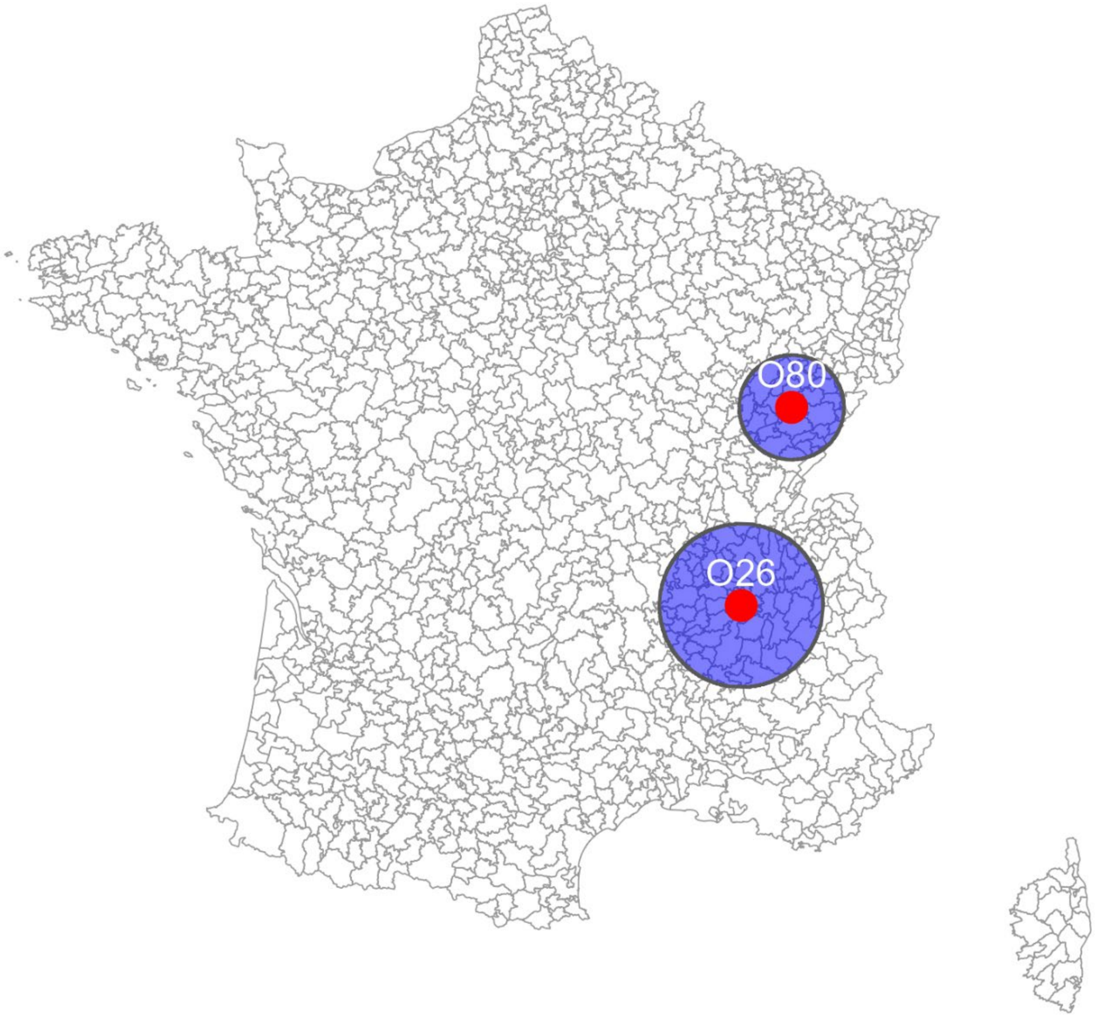

Appendix Figure 3. Significant clusters detected by space-time scanning by serogroup (O26, O80, O157) of sporadic Shiga toxin-producing *Escherichia coli*–associated pediatric hemolytic uremic syndrome cases reported in France over the entire 10-year period from 2012–2021.
